# Predicting the benefit of stereotactic body radiotherapy of colorectal cancer metastases

**DOI:** 10.1016/j.ctro.2022.07.006

**Published:** 2022-07-21

**Authors:** Sara Lindberg, Eva Onjukka, Peter Wersäll, Caroline Staff, Rolf Lewensohn, Giuseppe Masucci, Karin Lindberg

**Affiliations:** a Department of Oncology and Pathology, Karolinska Institutet, Stockholm, Sweden; b Section of Head, Neck, Lung and Skin Tumours, Theme Cancer, Karolinska University Hospital, Stockholm, Sweden; c Medical Radiation Physics and Nuclear Medicine, Karolinska University Hospital, Stockholm, Sweden; d Department of Radiotherapy, Karolinska University Hospital, Stockholm, Sweden; e Capio St. Göran Hospital, Stockholm, Sweden

**Keywords:** Stereotactic, SBRT, Colorectal, Metastases, Prediction, CLICAL© algorithm

## Abstract

**Aim:**

To evaluate Stereotactic body radiotherapy (SBRT) in metastatic colorectal cancer (mCRC) and identify the benefit of the treatment by using a predictive algorithm.

**Methods:**

85 patients treated with SBRT for mCRC were retrospectively analyzed. The CLInical Categorical Algorithm (CLICAL©) was used to predict probability of relapse after SBRT. Variables pre-SBRT were tested for significance for time to relapse (TTR). The patients’ CLICAL© score was the mean of sub-scores of each significant variable’s effect on the endpoint. Patients with similar scores were grouped into four signatures dependent on level of benefit after SBRT.

**Results:**

Median age was 69 years (42–88), 63 % had a performance status 0 and 47 % were treated for a single metastasis. At the time of the analysis, 90 % had relapsed (95 % out-of-field). Median TTR was 7.3 months (4.6–8.5), and the 2-year relapse-free rate was 15 % (95 %CI = 7–22). The CLICAL© signature III-IV predicted a low risk of relapse if receiving high dose SBRT to all metastases or to lung metastases only. Signature I-II had a short TTR, why SBRT for these patients was judged non-beneficial.

**Conclusion:**

The benefit from SBRT varies among mCRC patients. CLICAL© may serve as a screening tool for SBRT referrals but needs to be validated.

## Highlights

• Predicting the benefit from Stereotactic body radiotherapy (SBRT) of colorectal cancer metastases.
• CLInical Categorical Algorithm (CLICAL©) – a predictive algorithm applied to SBRT.
• The benefit from SBRT varies among patients with metastatic colorectal cancer.
• CLICAL© may be used as a screening tool for SBRT referrals.

## Background

Metastatic colorectal cancer (mCRC) is one of the leading causes of cancer-related deaths worldwide [1]. Approximately 25 % of the patients with CRC present with synchronous metastases and another 50 % will in the course of time develop metachronous metastatic disease [1], [2]. The liver is the most common metastatic site in colon cancers because of the venous drainage from the lower gastrointestinal tract [3], whereas rectal cancers are more likely to spread to the lungs through the hemorrhoid veins [4]. The organ site of metastatic spread in CRC is of prognostic significance [5], where patients with brain and bone metastases have poorer prognosis compared to patients with metastatic spread to the lung and liver [6].

Palliative chemotherapy is associated with a median overall survival (OS) of 30 months [2], [7] and a 5-year OS-rate of 14 % [1] and is the main oncological treatment for mCRC. However, for selected patients improvements in systemic therapies, such as new chemotherapy regimens, immunotherapy and targeted therapies, alone or in combination with local therapies may attain prolonged survival if treating the metastatic lesions locally to reduce the tumour burden while controlling the systemic disease spread with medical therapies [8], [9].

In terms of local treatment, surgery is the standard of care for patients with limited (oligometastatic) disease. The 5-year survival rate after resection of colorectal liver- or pulmonary metastases is 40–60 % [10], [11] and similar survival rates (40–50 %) have been reported after repeated resection of metastatic lesions at multiple sites [12], [13]. Patients unfit for surgery and with limited disease may instead be offered other ablative therapies, such as stereotactic body radiotherapy (SBRT) [14], [15] or ablation using radiofrequency (RFA) [16] or microwaves [17], with a resultant 3-year survival rate of 76 % [17].

SBRT is a non-invasive, high-precision radiation therapy technique that delivers ablative radiation doses. It is a well-established method for treating metastases in various organs (lung, liver, lymph node, adrenal gland, bone) [18], [19], [20], [21], [22] with high local control (LC) of the treated lesions (>80 %) and acceptable side effects [18], [23], [24]. SBRT of solitary colorectal liver- and pulmonary metastases may achieve LC-rates above90 % [24], [25], [26] and survival rates similar to pulmonary metastasectomy [27]. However, long term survival post SBRT of mCRC is generally inferior in comparison to surgery, which might at least partly be explained by patient selection in terms of worse performance status, comorbidities and frailty, and further by tumour-related factors such as synchronous metastases, greater tumour burden, unfavourable lesion location and larger tumour size [14], [27], [28]. Given these different considerations, it is of utmost importance to identify patient- and disease-specific parameters which optimally select mCRC-patients that benefit from SBRT, to avoid disproportionate side-effects and to promote health-economic care.

Individualized medicine, where evidence is used to select the best treatment for each patient, is an emerging field in clinical oncology. In mCRC, prognostic and predictive variables have been utilized in models to predict the outcome of a planned treatment for individual patients [29], [30], [31], [32], [33]. However, evaluating the benefit of SBRT for these patients may be challenging considering that patients with mCRC represent a heterogeneous patient group with different tumour characteristics where different combinations of systemic and local treatments are used to tailor the treatment for the patient. Hence, there is a great need for a tool to identify the patients who benefit from SBRT [28]. For this purpose, in this retrospective study applying a predictive algorithm (CLICAL©)[34], [35] to clinical data, we identify subgroups of patients with different outcome, in terms of TTR after SBRT of metastases from colorectal cancer.

## Methods

### Study cohort

All patients treated with SBRT for mCRC between January 2008 and August 2016 at Karolinska University Hospital were retrospectively included. In total, 85 patients were treated with 1–4 courses of SBRT. The analysis was based on the first course of SBRT, given the purpose to develop a screening tool. Patient- and tumour characteristics at the time of primary cancer diagnosis (tumour stage, histopathological subtype, number of CRC-primaries, treatment for primary tumour) and within 2 months of SBRT (performance status (PS), carcinoembryonic antigen (CEA), body mass index (BMI)) were retrieved from the medical records, as well as presentation of first metastatic disease (synchronous or metachronous metastasis), metastatic therapies given prior to SBRT (systemic or local), information regarding tumour progression post SBRT (radiological examinations every 3–6 months for 5-years after SBRT) and survival. SBRT-related toxicity was analyzed and graded according to the common terminology criteria for adverse events (CTCAE v.4.0) [36]. SBRT-treatment characteristics (number of targets treated, prescription dose, number of fractions) were retrieved from the treatment planning system (ARIA® oncology information system and Eclipse™ treatment planning system. Varian Medical Systems).

### SBRT-technique and assessment of radiotherapy data

The technique used for SBRT-immobilization and treatment planning has been described in detail in previous reports from our institution [37], [38]. In brief, in preparation for the SBRT-treatment a treatment-planning CT-scan was performed with the patient immobilised in the stereotactic body frame (Elekta AB, Stockholm, Sweden). During the study period, the SBRT-technique underwent some changes. The tumour motion was initially assessed by fluoroscopy (18 patients before 2011) which was later replaced by 4D-CT (67 patients after 2011). Abdominal compression was generally applied for abdominal- and lung targets for patients where, A) the diaphragm motion assessed by fluoroscopy exceeded 10 mm (old method), or B) the tumour motion assessed by 4D-CT exceeded 10 mm (new method). For treatment of tumours in the liver, a fiducial gold marker was implanted near the tumour for purposes of on-line matching. The clinical target volume (CTV) comprised the tumour with a margin of 1–2 mm accounting for the diffuse growth at the borders. Initially, the planning target volume (PTV) was determined by adding a 5–10 mm margin around the CTV. Since 2009, the margin was tailored to each patient if the tumour motion exceeded 10 mm, based on the amplitude of the tumour motion, estimated by 4D-CT. Relevant organs at risk (OAR) were delineated according to local guidelines. Tumour position was verified using online cone-beam CT (82 patients after 2009) or a verification CT (3 patients before 2009). The dose was prescribed to the 65–70 % isodose line encompassing the PTV with a resultant inhomogenous dose distribution and a maximum dose in the center of the target of about 1.5 times the prescribed dose; i. e a prescribed dose of 17 Gy × 3 = 51 Gy would yield a maximum dose in the centre of the target of about 25.5 Gy × 3 = 77 Gy. The treatment was delivered with 6 MV from a linear accelerator, using 5–10 static non-opposing coplanar fields (n = 70), or VMAT using 2–6 arcs (n = 15). As of year 2008 the AAA dose calculation algorithm was used, replacing Pencil Beam. For the analysis physical doses were converted into biological effective doses (BED) using the formula: BED = nd(1 + d/[α/β]), where n is the number of fractions, d is the dose per fraction, and the α/β-ratio for tumour was assumed to be 10 Gy (BED_10 Gy_).

The fractionation schedule 7 Gy × 8 (corresponding to 95 Gy in BED_10Gy_) was used frequently between 2010 and 2016 at our institution as a curative dose for centrally located lung lesions [38]. Thus, we defined curative SBRT-intention as a prescribed dose of ≥ 95 Gy in BED_10Gy_ to all known tumour lesions delivered with SBRT-technique, or a prescribed dose of ≥ 95 Gy in BED_10Gy_ with SBRT-technique to some metastases and another local ablative therapy to the rest of the known tumour burden to obtain ablation of all macroscopic tumour tissue. A palliative intent was identified where SBRT was prescribed at < 95 Gy in BED_10 Gy_ (regardless of number of metastases treated) or where not all metastases in the body were treated at the time of SBRT (regardless of prescribed dose).

### Statistical analysis

Time to relapse (TTR), LC and OS were modelled using the Kaplan-Meier method, where the start date was chosen as the start of the first course of SBRT. For TTR a statistical event was defined as any progression (local or distant) after SBRT and time to relapse was calculated until the date of the first radiologically verified progression. For LC a statistical event was defined as local progression defined as an increase of at least 20 % in the longest diameter of the treated lesion. Patients with no radiology performed after SBRT were not included in the analysis for TTR and LC. The survival time was calculated until death from any cause or the last date when the patient was known to be alive.

Following dichotomization or trichotomization of pre-SBRT clinical variables, their significance with regard to TTR was evaluated through a log rank test with p ≤ 0.05 considered statistically significant. The intervals of dichotomized or trichotomized variables were identified iteratively to achieve the greatest difference in TTR between the groups. All analyses were performed with StatView™ for Windows, SAS Institute Inc., Version 5.0.1.

The Clinical Categorical Algorithm (CLICAL©) [34], [35] was used to define categories of patients with similar TTR after SBRT. It consists of calculating a score based on the patient- and tumour related variables and grouping the patients with similar scores. These groups express different signatures for TTR, illustrated by Kaplan Meier survival- and cumulative hazard plots. The CLICAL© score is the mean of the sub-scores, which for each significant dichotomized or trichotomized variable takes the value of 1, 2 or 3, where 1 implies the greatest risk. The patient scores typically form clusters, and the number of signature groups were selected as the largest number associated with a significantly different TTR when comparing the resulting groups. Patients with missing data (for the significant variables included in CLICAL©), were not included in the CLICAL©-analysis.

The risk associated with SBRT-related variables was explored visually by stratifying the signatures for each variable in turn: the site of the treated metastases (only lung versus other) and treatment intent (curative versus palliative). As described above, curative intent was assumed when the total prescribed dose to the periphery of the PTV exceeded 95 Gy in BED_10Gy_, for all known active metastases in the body at time of SBRT-treatment.

## Results

### Patient-, tumour- and treatment characteristics

Eighty-five patients (54 % male) were included in the analysis; descriptive statistics are listed in Table 1. The median age at SBRT was 69 years (40–85 years), and 67 % of the patients had PS 0. Of the primary colorectal tumours (46 % colon), mismatch repair (MMR) deficiency were analyzed in 12 % (n = 10), mutations in RAS/RAF in 45 % (71 % of them were mutated), and histopathological differential grade in 95 % (10 high, 15 low, 54 medium–high grade). The majority of the primary tumours (90 %) had been surgically resected, 90 % of them were radical. Forty patients (47 %) presented with synchronous metastases. Prior to the SBRT- treatment, the average value of CEA was 208 µg/L (1–13194 µg/L) and 66 % of the patients had received therapy for metastatic control (out of which 28 % local treatment only, 16 % systemic treatment only, 22 % local and systemic treatments).

**Table 1:** Study cohort characteristics.

Patient characteristics		Number [range]	Percentage
Patients		85	100
Men		46	54
Age *(years, median)*		69 [40–85]	
Performance status: 0 / 1–2 / 3		57/27/1	67/32/1
**Tumor characteristics**			
Primary tumor: Colon/Rectum		44/50	46/54
Number of CRC-primaries 1 / 2 / 3		77/7/1	91/8/1
No analyzed for RAS/RAF-mutation		39	45
No tested positive		28	71*
Synchronous metastases**		40	47
Pre-SBRT: CEA-value < 5 µg/L		55	65
Pre-SBRT**: Treatment for metastatic control:		56	66
Local / Systemic / Local + Systemic		24/14/18	28/16/22
**SBRT treatment course 1**			
Total no of treated tumors		156	100
Pre-SBRT: No of active metastases in the body:			
1 / 2 / ≥3		21/24/40	25/28/47
No of metastasis treated per patient: 1 / 2 / 3 / 4		40/24/16/5	47/28/19/6
Metastatic site treated: Lung only		65	76
Fractionation schedule:	17 Gy × 3	118	75
	15 Gy × 3	15	10
	8 Gy × 5	5	3
	10 Gy × 3	3	2
	Other	15	10
Doses in BED (α/β 10, Gy): ≥ 95 Gy		76	89
All metastases treated		55	65
SBRT-indication: Curative^□^		50	59
**Outcome**			
Progression**^, □^^□^		76	90
TTR *(months, KM-est. median)*		7.3 [CI 95 %=4.6–8.5]	
2-year LC-rate *(%)*		88 [CI 95 %=80–90]	
Alive at last follow up		18	21
OS (*months, KM-est, median)*		30 [CI 95 %=20–36]	

* based on tumors tested for mutation. ** based on patient cohort. ^□^Curative = all active metastases treated with a BED_10_ ≥ 95 Gy.. ^□□^SBRT-doses between 48 and 132 Gy in BED_10_

Abbreviations: CRC: Colorectal cancer, CEA: Carcinoembryonic antigen, BED: Biological equivalent dose, TTR: Time to relapse, LC: Local control, OS: Overall survival.

In the first course of SBRT, 156 colorectal cancer metastases were treated in total and located in 4 different organs: lung (n = 134), liver (n = 15), lymph nodes (n = 6) and spine (n = 1). Forty-four percent of the patients were treated for a single metastasis, 76 % received treatment for lung metastases only and the most common fractionation schedule was 17 Gyx3 (75 % of the treated lesions). Fifty-nine percent received SBRT with curative intent.

The side effects were evaluated throughout the follow-up time, sometimes including follow-up after subsequent SBRT treatment courses, since late effects from the first treatment course may appear after further treatment has been received (the number of courses received were: 85 patients - 1 course, 28 patients – 2 courses, 5 patients – 3 courses and 2 patients – 4 courses). The treatment was generally well tolerated; overall toxicity is presented in Table 2. The most commonly reported treatment related side-effect was radiation pneumonitis which was scored in 11 patients (13 % of the patient cohort): 1 grade 4, 3 grade 3 and 7 grade 2. In total, three grade 4 side-effects occurred after SBRT (gastrointestinal bleeding, respiratory failure, radiation pneumonitis); no grade 5 event was seen.

**Table 2:** Overall toxicity after SBRT course 1–4.

	Grade				
Adverse event	1	2	3	4	Total
Dyspnea		2			2
Fibrosis deep connective tissue	1				1
Gastro-intestinal-bleeding				1	1
Pain in thorax	1	4	1		6
Radiation pneumonitis		7	3	1	11
Respiratory failure				1	1
Rib-fracture		2			2

### Outcome: Time to relapse, local control, overall survival

**Fig. 1:**
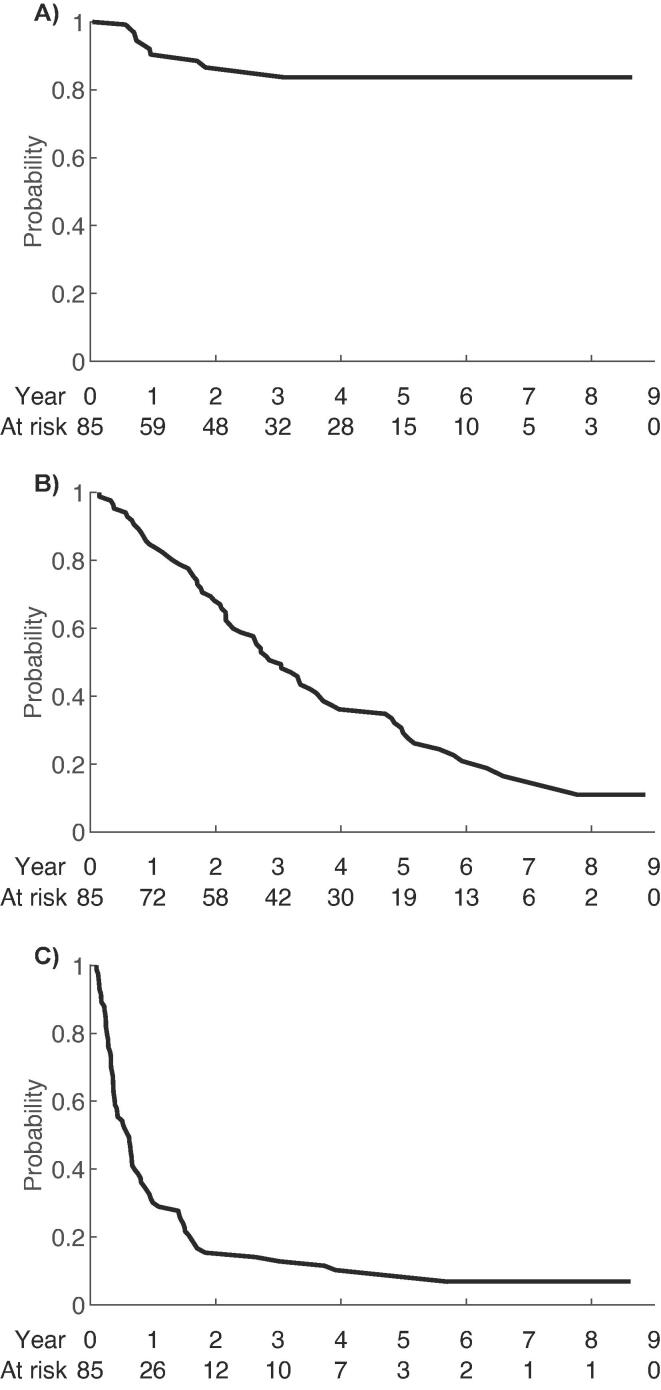
Kaplan-Meier curves illustrating the probability of local control (A), overall survival (B) and relapse (C) after SBRT of colorectal cancer metastases.

At last follow up, 90 % of the patients had relapsed after SBRT and 79 % of the patients were deceased.The first relapse was more likely to occur outside the irradiated field (n = 76) as compared to in-field failure (n = 4). Median TTR was 7.3 months (4.6–8.5 months) and relapse-free rate at 6 months, 1-, 2- and 5-years were 55 % (95 % CI = 45–66 %), 30 % (95 % CI = 21–40 %), 15 % (95 % CI = 7–22 %) and 3.5 % (95 % CI = 0–7 %) respectively. LC-rates at 1-, 2- and 5-years were 92 % (95 % CI = 85–88 %), 88 % (95 % CI = 80–98 %) and 86 % (95 % CI = 76–95 %). Local failure after SBRT occurred in 8 patients treated for 9 tumours: 4 in liver (BED_10Gy_ = 58–106 Gy) and 5 in lung (BED_10Gy_ = 72–138 Gy). Median OS after SBRT was 36.5 months (2–106 months) and survival rates at 6 months, 1-, 2- and 5-years were 95 % (95 % CI = 90–100 %), 80 % (95 % CI = 70–90 %), 70 % (95 % CI = 60–80 %) and 29 % (95 % CI = 20–40 %). Kaplan-Meier analyses are presented in Fig. 1A-C.

In the log rank test, the significant pre-SBRT variables (p ≤ 0.05) for TTR were age, PS, number of CRC-primaries, CEA-value and number of active metastases in the body prior SBRT. Table 3 lists the p-values from the logrank test and the univariate and multivariate analyzes is listed in Supplementary table 1.

**Table 3:** Variables tested with log rank test (p-values) for time to relapse after SBRT and the received weights for the significant variables (p ≤ 0.05) included in the CLICAL© algorithm. A) Variables with p ≤ 0.05 in log rank test and included in the algorithm. B) Variables with p > 0.05 in log rank test.

Variables	Log rank test		CLICAL© weight
	Log rank (χ^2^)	*p*	1/2/3
Age, years			
<65 vs ≥ 65	5.79	0.01	1/2
PS			
≥2 vs 0–1	3.66	0.05	1/2
Number of CRC-primaries			
≥2 vs 1	18.55	0.001	1/2
CEA, µg/L			
≥10 vs 6–9 vs < 5	4.98	0.02	1/2/3
Number of active metastases in the body before SBRT			
≥3 vs < 3	18.55	0.0001	1/2

Variables	Log rank test	
	Log rank (χ^2^)	*p*
Gender		
male vs female	0.002	0.98
Treatment prior SBRT ^¤^		
systemic ± local vs local or none	1.25	0.26
BMI, kg/m^2^		
≥ 30 vs < 30	0.26	0.60
Radical surgery of primary tumor		
no vs yes	2.09	0.14
Primary tumor *		
rectum vs colon	0.47	0.49
Differential grade (primary tumor) *		
high vs medium–high vs low	1.47	0.47
Metastatic disease		
synchronous vs metachronous	0.06	0.80
Local therapies prior SBRT ^¤^		
0 vs 1 vs ≥2	0.66	0.71
Systemic cycles prior SBRT ^¤^		
0–1 vs ≥2	1.6	0.20

Abbreviations: CLICAL©: Clinical categorical algorithm, PS: Performance status, CRC: Colorectal cancer, CEA: Carcinoembryonic antigen.

Abbreviations: BMI: Body mass index, CLICAL©: Clinical categorical algorithm, vs=versus.

For metastatic disease. *If multiple colorectal cancers, the first being diagnosed.

### CLICAL© analysis

The CLICAL© analysis for TTR was performed on 79 patients (6 patients excluded due to missing data), stratified in four sub-groups with significantly different outcome (signature I score interval = 1.2–1.4, signature II score interval = 1.6–1.8, signature III score = 2.0, signature IV score = 2.2). The number of patients in each subgroup was: 11 with signature I, 31 with signature II, 27 with signature III and 10 with signature IV. The distribution of risk-factors in each signature (I-IV) is illustrated in Supplementary table 2.

**Fig. 2:**
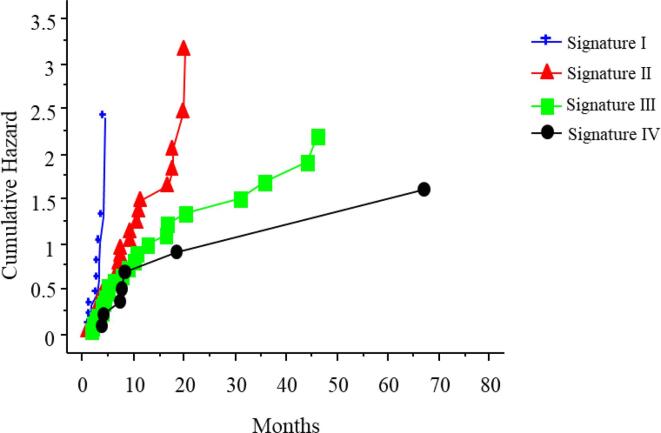
Patients with mCRC categorized in four subgroups (signature I-IV) according to their benefit, in terms of time to relapse (TTR) after SBRT using the Clinical Categorical Algorithm (CLICAL©); signature I (blue, n = 11), signature II (red, n = 31), signature III (green, n = 27) and signature IV (black, n = 10). Signature I and signature IV imply the smallest and greatest benefits from SBRT respectively (p ≤ 0.05)**.**

The signatures indicate the benefits after SBRT, where signature I and IV imply the smallest and greatest benefits respectively (Fig. 2). Given the difference in the time to 50 % risk of relapse between signature I (3 months) and signature IV (8 months) it is apparent that patients with low signatures did not benefit clinically from SBRT.

**Fig. 3:**
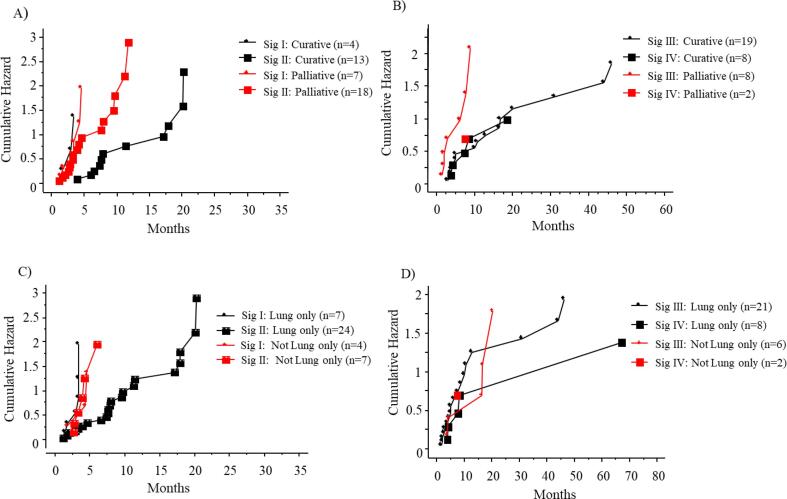
Time to relapse after SBRT for signature I-II versus III-IV when stratified for SBRT-intention; curative versus palliative (A, B) and site of the treated metastases; lung only versus other (C, D). Curative intent was defined as all active metastases treated with a total prescribed dose to the periphery of the PTV ≥ 95 Gy in biologically equivalent dose (BED) using α = 10 Gy.

When stratifying for SBRT-related variables, we found that signature III-IV predicted a low risk of relapse if receiving curatively intended SBRT, with 50 % risk of relapse after 8 months in contrast to after 3 months for patients with palliative intent (Fig. 3A-B). The risk of relapse was lower in all signatures if only lung metastases were treated, with 50 % risk of relapse after 6–8 months, compared to 3–6 months for other patients (Fig. 3C-D).

## Discussion

The results of this study show that when applying CLICAL© to pre-SBRT clinical variables, patients with mCRC can be categorized into subgroups according to their benefits from SBRT, with potential to obtain a long relapse-free time. The CLICAL© algorithm might thus be useful as a screening tool for SBRT-referrals.

In our study cohort the majority of the patients (95 %) had an out-of-field recurrence as first relapse after SBRT, which is in line with previous reports after SBRT of mCRC [39], [40]. This finding, in combination with our high rate of LC throughout the follow-up period (2-year LC-rate = 88 %), indicate great effectiveness of SBRT as local treatment. This observed pattern of relapse after SBRT for these patients is of interest since it reflects that relapses most often may contribute to a systemic failure due to uncontrolled systemic spread of the disease rather than to poor performance of SBRT. It also highlights the clinical challenge which consists of selecting patients with an anticipated long relapse-free systemic survival as candidates for SBRT.

Interestingly, the location of the SBRT-treated metastasis seems to influence the risk of systemic spread, as 50 % of patients in all signatures who received SBRT for lung metastases only, had no relapse at 8 months post SBRT, as compared to patients treated for extra-pulmonary metastases where 50 % of the patients had relapsed after approximately 3–6 months post SBRT. These results are consistent with results from previous studies evaluating SBRT of liver- and pulmonary mCRC, showing better response after SBRT of pulmonary metastases compared to metastases located extrapulmonary [41], [42]. Similarly, a higher OS rate is reported after SBRT of cases with CRC-pulmonary metastases as compared to SBRT of extrapulmonary metastases [43]. The different responses after SBRT, may be a consequence of the extrapulmonary location implying a less indolent cancer type with faster growing metastases [44] or of a more advanced tumour spread with extensive subclinical disease compared to pulmonary metastases only.

Reported local control rates after SBRT of CRC-metastases in the liver have been somewhat inferior as compared to those after SBRT of pulmonary metastases, with 2-year LC residing between 73-91 % for liver metastases in comparison to between 92-100 % for metastases located in the lung [41], [45]. Inferior LC-rates are also reported after SBRT of pulmonary metastases originating from CRC compared to SBRT of lung metastases from other primaries [43], [46], which may be a result of increased radio resistance related to hypoxia [40]. In mCRC, some evidence even suggests that lung metastases from colon cancer may be even more resistant than lung metastases from rectal cancer [46], [47]. The increased radio-resistance in mCRC may however be overcome by dose escalation to the target [48], and even though the optimal dose for these metastases has not yet been established, centres tend to use higher fraction doses to overcome the resistance [46], [48]. In the current study we used a median prescription dose of 138 Gy in BED_10Gy_ (75 % of the treated lesions) prescribed to the periphery of the PTV, resulting in a central dose in the target of approximately 207 Gy (BED_10Gy_), which resulted in a 2-year LC-rate of 88 %. The LC-rates reported in this study is comparable to the reported 1- and 2-year LC-rate of 90 % in studies evaluating SBRT of CRC-metastases with comparable prescription doses [42], [47], [49].

The median survival post metastasectomy of hepatic- and pulmonary CRC-metastases ranges between 40 and 60 months and the 1- and 2-year survival is about 95–97 % and 80–85 % respectively [10], [11] and are thus generally superior to those reported post SBRT [24], [25], [26], [49], [50]. Despite both being radical treatments, survival outcome after metastasectomy and SBRT may in this situation be difficult to compare since patients who are not candidates for surgical intervention may be compromised with comorbidities or have technically unresectable metastases. The disease may also be in a more advanced stage [51] with shorter life expectancy of the patient. However, in comparison to studies both of metastasectomy and of SBRT with more favourable patient characteristics (PS 0–1) and limited CRC-metastases (oligometastases) [39], [49] our study reports comparable 1- and 2-year survival-rates of 80 % (95 % CI = 70–90 %) and 70 % (95 % CI = 60–80 %), which is encouraging. To evaluate survival time after any local treatment for metastases is, however, challenging considering the possible influence of other local and systemic treatments. Despite this, OS is still an important contextual measurement when evaluating the benefits of the local treatment.

The number of metastases in the body at time of SBRT can be correlated with the TTR after SBRT of mCRC [39], [52], [53], [54]. In this study we found that patients with < 3 metastases prior SBRT had longer relapse-free time after SBRT, compared to patients treated with ≥ 3 metastases. Other studies also report that limited number of metastases (oligometastases), generally between 1 and 5 metastases, results in longer relapse-free time after SBRT [39], [53], [54] compared with those showing > 5 metastases. Interestingly, the number of metastases in the body at time of given local treatment may also be correlated with the pattern of progression, where patients with < 3 metastases are more likely to relapse with a limited number of metastases, amenable for further metastasis-directed therapy, while patients with > 3 metastases, that are more likely to relapse with a widespread disease, require a switch to systemic therapy [54].

Franzese et al. investigated predictive factors for survival post SBRT in a retrospective study of SBRT of 270 patients treated for 437 CRC-oligometastases located in the liver or the lung and concluded that a target size of >30 mm, extrapulmonary disease and systemic treatment prior SBRT all predicted worse OS [55]. Timing of the first metastatic presentation in CRC (synchronous or metachronous) metastatic presentation) is reported of prognostic significance in CRC [56], [57]. Possibly, this relates to the differences in pathologic characteristics and angiogenesis [56] as well as to the clinical presentation, where greater tumour burden is reported for synchronous metastases [56], [57], [58]. In this study we did not find any significant correlation between the time point of first metastatic presentation and TTR after SBRT. Interestingly, we found that multiple CRCs at primary diagnosis [59], [60] is of prognostic value for the risk of recurrences after SBRT of mCRC. Additionally, CEA-levels prior to SBRT seem to influence the outcome. This is interesting since the use of the CEA-levels in predicting response to chemo- and radiotherapy is controversial, as some studies show no correlation [61], whereas other indicate that a higher CEA-value correlates with poor response [62]. The limited reliability of the CEA-values may be a plausible explanation, since false negative values may be observed in poorly differentiated colorectal cancer types [63], while at the same time, elevated values may be associated to other factors such as non-colorectal malignancies, smoking, cirrhosis and inflammations [63], [64].

Genetic tumour alterations such as mutations in the RAS/RAF and the MMR-genes are reported to be of prognostic and predictive value in mCRC [65] and are found more frequently in younger patients [66], [67]. In this study the influence of mutational burden could unfortunately not be tested since these analyses were not in clinical routine at the time of the study, resulting in only a small number of patients being tested. Similarly, the potential influence of laterality of the primary tumour for colon cancer (n = 44), which is a known prognostic factor [68], could not be analyzed due to the small patient cohort. However, we noticed a higher risk to relapse after SBRT in younger patients (<65 years) compared to older (≥65 years), which may be explained by the underlying genetic and sporadic alterations affecting the aggressiveness of the tumour and risk of recurrences [66].

Prediction models evaluating clinical risk factors for the development of metastatic disease and recurrences of colorectal cancer do exist [30], but prediction models evaluating SBRT for mCRC are less common. Ji et al [39], created a nomogram to predict the probability of survival post SBRT of mCRC based on retrospective data from 94 patients who had been treated with SBRT for 162 colorectal metastases. Some of the chosen variables were similar to those in our model (PS, pre-SBRT CEA-value and the number of metastases treated), but they also included the PTV-size as well as the SBRT treatment indication (oligometastases, oligoprogression or local control of dominant tumours). The median progression-free survival (PFS) for the whole cohort was 7.0 months (95 % CI = 4.9–9.1 months) and for the respective subgroups based on the treatment indications, oligometastases, oligoprogression or LC of dominant tumours, the median PFS-rates were 12.6 months, 6.8 months, and 3.7 months, and correspondingly values for OS were 40.0 months, 26.1 months, and 6.5 months [39]. Similar as the study by Ji et al, we analysed the PTV-size of treated metastasis. However, since a combined PTV-volume for patients treated for multiple metastases may represent different disseminated metastatic disease (one single large lesion compared to many small lesions at multiple sites) which might affect the outcome, we decided not to use the variable as a measure of benefit from SBRT. Like the previously proposed nomogram by Ji et al, the CLICAL© algorithm is convenient to use and based on available clinical variables. A particular strength of the current model is that it takes into account the importance of SBRT treatment parameters. As indicated by our results (Fig. 3A-B), treating all known metastatic lesions as well as maintaining a prescription dose of at least 95 Gy BED_10Gy_ to all known tumour lesions seems important for the aim of postponing progression and perhaps prolong survival, while still being a safe treatment with tolerable side-effects.

We consider TTR the best parameter to measure the benefit from SBRT in this group of patients, with metastatic colorectal cancer. The OS will be influenced by the patient’s previous medical history and other systemic and local therapies used to control the cancer disease before and after SBRT. On the other hand, LC after SBRT measures the effectiveness of the SBRT-treatment to the treated lesion, hence not the overall benefit to a patient in the metastatic state. Furthermore, LC, which not necessarily reflect the overall benefit of the treatment, is difficult to evaluate in a retrospective cohort as the radiological examinations may not be performed per routine and each lesion may not be evaluated in detail if the disease is disseminated.

The retrospective design, the small patient cohort and missing data are limitations of our study that should be taken into consideration when interpreting the results. We could not evaluate the patients with regard to their treatment indication, which is a limitation since inferior survival and LC have been reported for patients treated due to oligoprogression and for locally controlling the tumour when compared to the indication oligometastases [39]. Similarly, we did not have access to translational information on gene expression profiling of all tumours and the radiological follow-up was not standardized given that all patients had metastatic spread. Even though the patients included in the analysis were consecutively treated, the results are based on patient data from only one institution implying a risk of selection bias of the patients and overfitting of the model. This is a clear limitation and warrants the model to be validated, preferably both in an external patient cohort as well as in a prospective setting.

## Conclusions

In this study we report SBRT as a safe and effective local treatment for the investigated group of patients. We found that age, PS, number of CRC-primaries, CEA-value and number of active metastases in the body prior SBRT to be of importance for the risk of relapse after SBRT. Based on the CLICAL© algorithm, we could also define a subgroup of patients for whom the treatment most likely will be highly beneficial and potentially result in a long relapse free time. Further, the algorithm highlighted the importance of treating all known metastatic lesions to a prescription dose of at least 95 Gy (BED_10Gy_). The CLICAL© algorithm may be of high value to indicate a patient’s probability of TTR after SBRT and could serve as decision support for SBRT-referrals. However, before taking the CLICAL© algorithm into the clinic, it needs to be validated in an external patient cohort.

## Declaration of Competing Interest

The authors declare that they have no known competing financial interests or personal relationships that could have appeared to influence the work reported in this paper.
