# Supplementary material for: Predicting the benefit of stereotactic body radiotherapy of colorectal cancer metastases
Source: Clin Transl Radiat Oncol. 2022 Jul 21;36:91–8. doi: 10.1016/j.ctro.2022.07.006 (PMC9356237; doi:10.1016/j.ctro.2022.07.006)
Supplement: Supplementary data 1 [file mmc1.pdf]

A)

Variables	UVA			MVA		
	HR	CI 95%	*p*	HR	CI 95%	*p*
**Age,** years						
<65 vs ≥65	0.43	0.22 - 0.8	0.008	0.59	0.35 - 0.99	0.04
**PS**						
≥2 vs 0 - 1	2.19	0.96 - 4.62	0.05	2.34	0.96 - 5.9	0.06
**No of CRC-primaries**						
≥2 vs 1	2.80	1.3 - 5.9	0.007	0.36	0.15 - 0.88	0.02
**CEA,** µg/L						
≥10 vs 6-9 and	0.84	0.35 - 2.0	0.69	0.74	0.27 - 1.9	0.55
≥10 vs ≤5	0.54	0.20 - 1.0	0.05	0.64	0.30 - 1.36	0.24
**No. of active metastases in the body before SBRT**						
≥3 vs <3	2.1	1.35 - 3.4	0.001	2.3	1.39 - 3.88	0.001

Abbreviations: UVA: Univariate analysis. MVA: Multivariate analysis. PS: Performance status. No: Number. CRC: Colorectal cancer. CEA: Carcinoembryonic antigen.

B)

**Variables**	**UVA**		
	HR	CI 95%	*p*
**Treatment prior SBRT**			
systemic ± local vs local or none	0.73	0.46 - 1.16	0.18
**BMI,** kg/m^2			
≥ 30 vs < 30	0.77	0.40 - 1.4	0.43
**Radical surgery of primary tumor**			
no vs yes	1.4	0.64 - 3.1	0.39
**Primary tumor**			
rectum vs colon	0.85	0.54 - 1	0.49
**Differential grade (primary tumor)***	0.64	0.29 - 1.4	0.27
high vs medium-high vs low	1.11	0.62 - 1.9	0.71
**Metastatic disease**			
synchronous vs metachronous	1.06	0.67 - 1.66	0.80
**Local therapies prior SBRT¤**	0.77	0.41 - 1.45	0.42
0 vs 1 vs ≥2	0.86	0.50 - 1.47	0.59
**Systemic cycles prior SBRT¤**			
0 - 1 vs ≥2	0.65	0.33 - 1.27	0.21

Abbreviations: UVA: Univariate analysis, BMI: Body mass index.
¤ For metastatic disease. *If multiple colorectal cancers, based on the first being diagnosed.
