# Supplementary material for: Predicting the benefit of stereotactic body radiotherapy of colorectal cancer metastases
Source: Clin Transl Radiat Oncol. 2022 Jul 21;36:91–8. doi: 10.1016/j.ctro.2022.07.006 (PMC9356237; doi:10.1016/j.ctro.2022.07.006)
Supplement: Supplementary data 2 [file mmc2.pdf]

	SIG I	SIG II	SIG III	SIG IV
**No. of patients**	11	31	27	10
**Age**, years				
≥65	6	13	21	10
<65	5	18	6	0
**PS**				
≥2	4	2	1	0
0-1	7	29	26	10
**No. of CRC-primaries**				
≥2	3	3	2	0
1	8	28	25	10
**CEA**, μg/L				
≥10	10	5	0	0
6-9	1	5	1	0
<5	0	21	26	10
**No. of active metastases at time of SBRT**				
≥3	9	20	10	1
<3	2	11	17	9

Abbreviations: Sig: Signature, No: Number, PS: Performance status, CRC: Colorectal cancer, CEA: Carcinoembryonic antigen. *Totally 6 patients excluded in the analysis.
